# Leveraging the CORE Group Partners Project Polio Infrastructure to Integrate COVID-19 Vaccination and Routine Immunization in South Sudan

**DOI:** 10.9745/GHSP-D-23-00178

**Published:** 2024-02-20

**Authors:** Anthony Kisanga, Kathy Vassos Stamidis, Samuel Rumbe, Doris Lamunu, Adil Ben, Gena Ruocco Thomas, Jean Berchmans

**Affiliations:** a CORE Group Partners Project South Sudan, Juba, South Sudan.; b CORE Group Partners Project, Washington, DC, USA.; c Johns Hopkins Bloomberg School of Public Health, Baltimore, MD, USA.

## Abstract

**Introduction:**

Countries with fragile health systems like South Sudan experienced significant impacts on routine immunization during the COVID-19 pandemic. Routine immunization in children aged younger than 1 year declined due to pandemic-related constraints and was compounded by the introduction of the COVID-19 vaccine, which was met with hesitancy and reluctance. When South Sudan reported the first COVID-19 case in March 2020, the CORE Group Partners Project (CGPP) rapidly integrated the COVID-19 outbreak response into its ongoing polio eradication activities, leveraging the existing polio infrastructure and human resources. We describe the integration process, results, and challenges and detail the impact of the integration on coverage for both routine immunization and COVID-19 vaccinations.

**Methods:**

Efforts to integrate COVID-19 vaccination and routine immunization service delivery were implemented in 5 phases: assessing the need, developing multisector collaborations, developing a service delivery plan, assessing implementation readiness, and implementing and evaluating the service delivery plan. Integration efforts prioritized coordination, training vaccinators and volunteers, development of microplans, data management, and last-mile vaccine delivery. Integrated service delivery was implemented through “one-stop shop” sessions where communities accessed routine immunizations for children, COVID-19 vaccinations for adults, and other primary health services.

**Results:**

Integrating health service delivery contributed to improved routine immunization coverage among children, improved COVID-19 vaccination coverage among adults, reduced cost for service delivery, and increased access to more comprehensive health services in hard-to-reach communities. COVID-19 vaccinations were delivered at US$4.70 per dose, a cost substantially lower than other reported delivery mechanisms.

**Conclusion:**

Integration can yield positive results and improve access to vaccination and other health services for communities. However, it requires clear policy guidelines, commitment, and strong collaboration. Challenges included resistance from stakeholders, overstretched human resources, and diversion of funding and attention from program areas, which were overcome through deliberate high-level advocacy, partnership, and intensified community engagement.

## Abstract (precis)

The article provides an adaptable model for resource-constrained settings for effectively integrating health care programs by showcasing how routine immunization can integrate with emerging disease prevention.

## Teaser

**Key Findings:**

- Integrating service delivery allows communities to access more needed services but is labor intensive and requires extensive collaboration and partnership.
- Integrating COVID-19 vaccination with routine immunization significantly increased vaccination coverage in South Sudan for both.
- Using the already established polio infrastructure and personnel made immunization services more accessible, cost-effective, and efficient.
- Involving trained health workers and volunteers as part of the community-based approach played a crucial role in eradicating vaccine fear and misinformation.
- Sharing resources and using existing networks eliminated duplicate human and material resources and led to cost savings and efficiency.

**Key Implications:**

- Policymakers can use these findings to support the development of integrated health policies to promote multisectoral collaboration and strengthen the resilience of health systems.
- Governments can use this potent strategy to simultaneously address multiple health challenges and enhance overall immunization coverage and access to health services for vulnerable communities.

Plain language article summary available.

## INTRODUCTION

The world’s newest nation, South Sudan, is home to approximately 11 million people.^1^ Since its founding in 2011, conflict has devastated health infrastructure, increased poverty, and made it difficult for communities to access primary health care services. The performance and capacity to deliver essential health services in South Sudan have been undermined by conflicts in all 3 tiers of governance and service delivery: national, state, and county levels responsible for tertiary, secondary, and primary health care.^4^^–^^7^ The state of the health care system in South Sudan is dismal. The availability of trained health workers is estimated to be just 3.5 per 10,000 population.^11^ Most of the population (90%) lives in rural areas, located substantial distances from health services and being consistently impacted by natural disasters and insecurity.

The 2018 revitalized agreement for the resolution of conflict in South Sudan brought about some gains in health sector recovery. However, these gains were set back by the COVID-19 pandemic, which disrupted the provision of essential health services, including surveillance and routine immunization (RI) for vaccine-preventable diseases (VPDs) in children aged younger than 1 year, hence putting many children at risk of VPDs.^2^^,^^3^

A World Health Organization (WHO) report indicated that RI programs in 68 countries had been disrupted by the COVID-19 pandemic, affecting more than 80 million children worldwide.^8^ The COVID-19 pandemic disrupted childhood vaccination so significantly that infant immunization decreased globally from 90% in 2019 to 86% in 2021,^9^ indicating that 25 million children aged younger than 1 year did not receive basic vaccines. Of those 25 million, 11 million reside in fragile or conflict-affected regions and 18 million are considered zero-dose—or unvaccinated, not having received any RIs. Disturbingly, this is the highest number of zero-dose children since 2005, representing 6 million more zero-dose children than before the pandemic. Sixty-two percent of zero-dose children live in 10 countries in Africa and Southeast Asia.^10^

In South Sudan, access to and uptake of RI was challenging before the COVID-19 pandemic and even more so after. Canceled immunization sessions, movement restrictions, and fear of COVID-19 infection worsened RI coverage. South Sudan experienced decreased numbers of children routinely vaccinated following the COVID-19 outbreak.^12^ A 2020 community-based cross-sectional study in rural communities in 4 counties of South Sudan’s Western Lakes State found that 75.5% of children were not following the prescribed immunization schedule.^13^ The RI schedule in South Sudan covers children aged younger than 1 year but has expanded to children up to age 23 months to account for significant population movement. Additionally, RI for women of childbearing age provides vaccines for tetanus and diphtheria. The population of zero-dose children increased during the COVID-19 pandemic and created a significant risk for polio outbreaks and other VPDs. RI is crucial to preventing not only VPDs but also global pandemics. Therefore, there was a need to leverage existing polio and RI infrastructure to integrate COVID-19 vaccination to increase vaccine uptake.

In South Sudan, access to and uptake of RI was challenging before the COVID-19 pandemic and even more so after.

COVID-19 vaccination in South Sudan began in April 2021, initially vaccinating health care workers and the elderly before expanding to include all adults aged older than 18 years.^14^ The vaccine was met with skepticism.^15^ Myths and misconceptions about COVID-19 and the newly introduced vaccine, including those about safety, risk, conspiracy theories, and health implications, permeated communities and led to low uptake.^16^ Vaccination rates among women were particularly concerning as myths about fertility issues linked to vaccines spread quickly. Hesitancy also began to affect RI choices.^17^ Community health programs had to quickly pivot to understand and address the drivers of vaccine hesitancy and uptake. Similar challenges were experienced in the fight to improve oral polio vaccine (OPV) uptake through the global polio eradication efforts.^18^ The lessons learned, vaccine infrastructure, and community networks built through polio eradication efforts provided a framework to build upon for COVID-19 vaccination programming.

## CORE GROUP PARTNERS PROJECT IN SOUTH SUDAN

The CORE Group Partners Project (CGPP), formerly known as the CORE Group Polio Project, initiated its work in South Sudan in 2010 to support the Ministry of Health (MOH) in eradicating polio, enhancing human resource capacity, and establishing structures for fortifying immunization systems and community-based disease surveillance (CBS). Since then, CGPP has built community structures to support polio eradication through a network of incentivized community health workers (home health promoters) and volunteers (community key informants). CGPP also provides technical support to strengthen immunization service delivery, build local partner capacity, and deploy risk communication and community engagement (RCCE) techniques, which include sensitization through visits to homes and social gathering places, advocacy meetings with community influencers, and focus group discussions on COVID-19 myths. To contribute to the Global Polio Eradication Initiative, CGPP focuses on hard-to-reach and conflict-prone regions of South Sudan, defined as^19^:

*Areas may become access-compromised due to conflict and insecurity, or they may be hard-to reach due to geographical barriers such as difficult terrain and poor roads. When geographical barriers or security issues impede access entirely, such areas and the people living within them become inaccessible.*

In South Sudan, insecurity, poor infrastructure, and geographical barriers often impede access to vaccine services. Although conflict has increased in South Sudan since the data collection of this study, in CGPP implementation areas, conflict has not escalated significantly enough to block project implementation. The major conflict continues to be cattle raiding, bandits, and ethnic clashes, each of which interrupts smooth implementation. CGPP continues to monitor security situations closely and adjust programming and community outreach as needed to ensure the safety of staff, volunteers, and communities.

**FIGURE 1:**
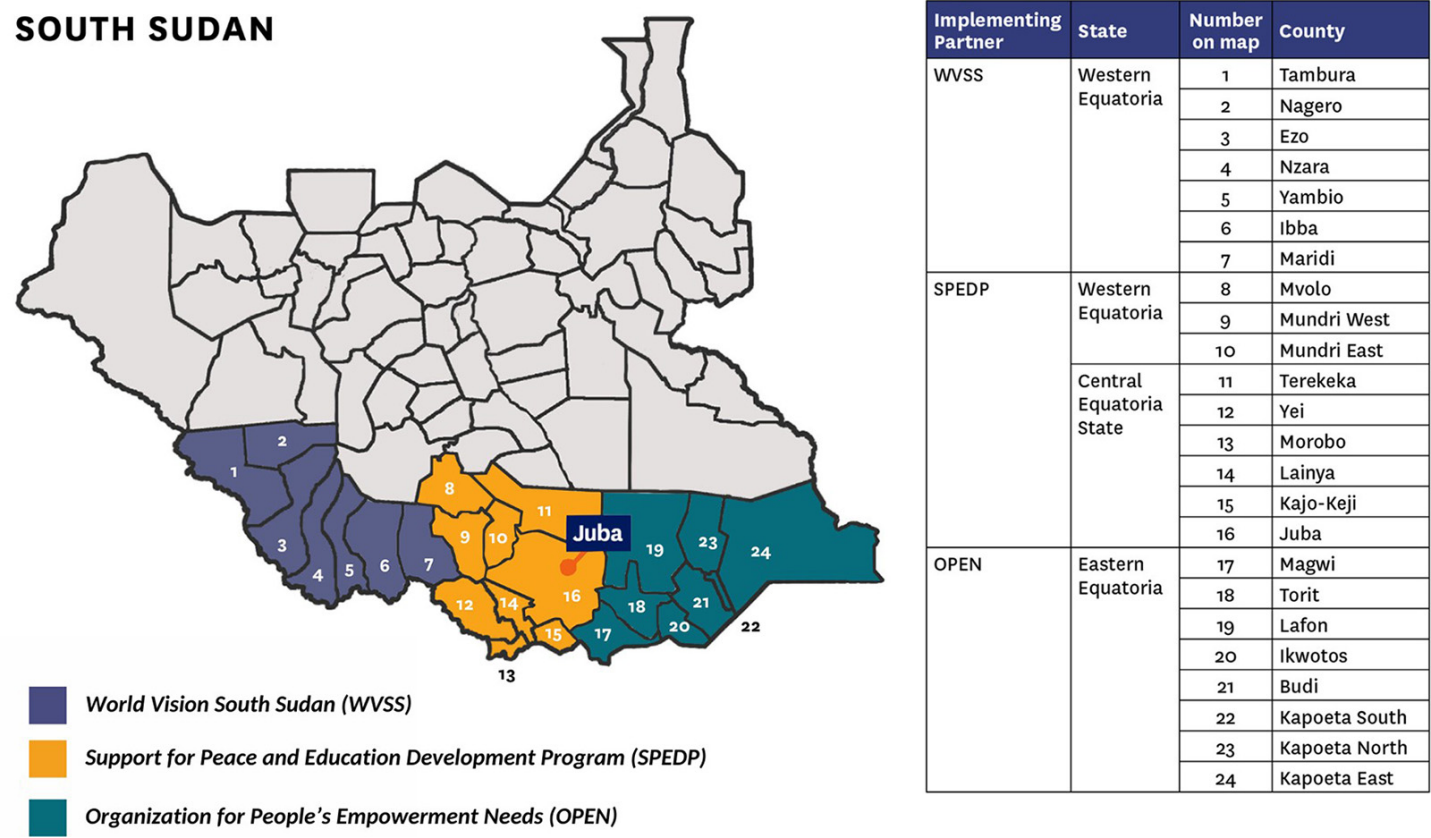
Map of Core Group Partners Project South Sudan’s Implementation Areas

CGPP currently operates in the southern part of South Sudan, specifically in the Equatoria Region, covering all 24 counties of Central Equatoria, Eastern Equatoria, and Western Equatoria states, where the intended population for social mobilization, health education, and COVID-19 vaccination is 1.96 million people aged 18 years and older. The population of children aged younger than 1 year eligible for RI is 178,452.^20^ The work is implemented through World Vision and 2 local partner organizations, Support for Peace and Education Development Program and Organization for People’s Empowerment Needs (Figure 1). Each partner operates in specific counties, creating effective partnerships and representation with government counterparts at subnational levels; organizing and facilitating trainings for mobilizers, vaccinators, and supervisors; conducting focus group discussions, advocacy meetings, and community dialogues; deploying vaccination teams and supervision; distributing last-mile vaccines; and documenting and reporting activities. Current CGPP activities in this region include CBS for VPDs and COVID-19, support for immunization campaigns, vaccination service delivery, RCCE, and, most recently, COVID-19 response. CGPP focuses on providing children aged younger than 1 year with vaccines for polio and other VPDs, including OPV0, OPV3, pentavalent third dose (Penta 3), and measles, and women of reproductive age (including pregnant women) with tetanus/diphtheria vaccine.

**FIGURE 2:**
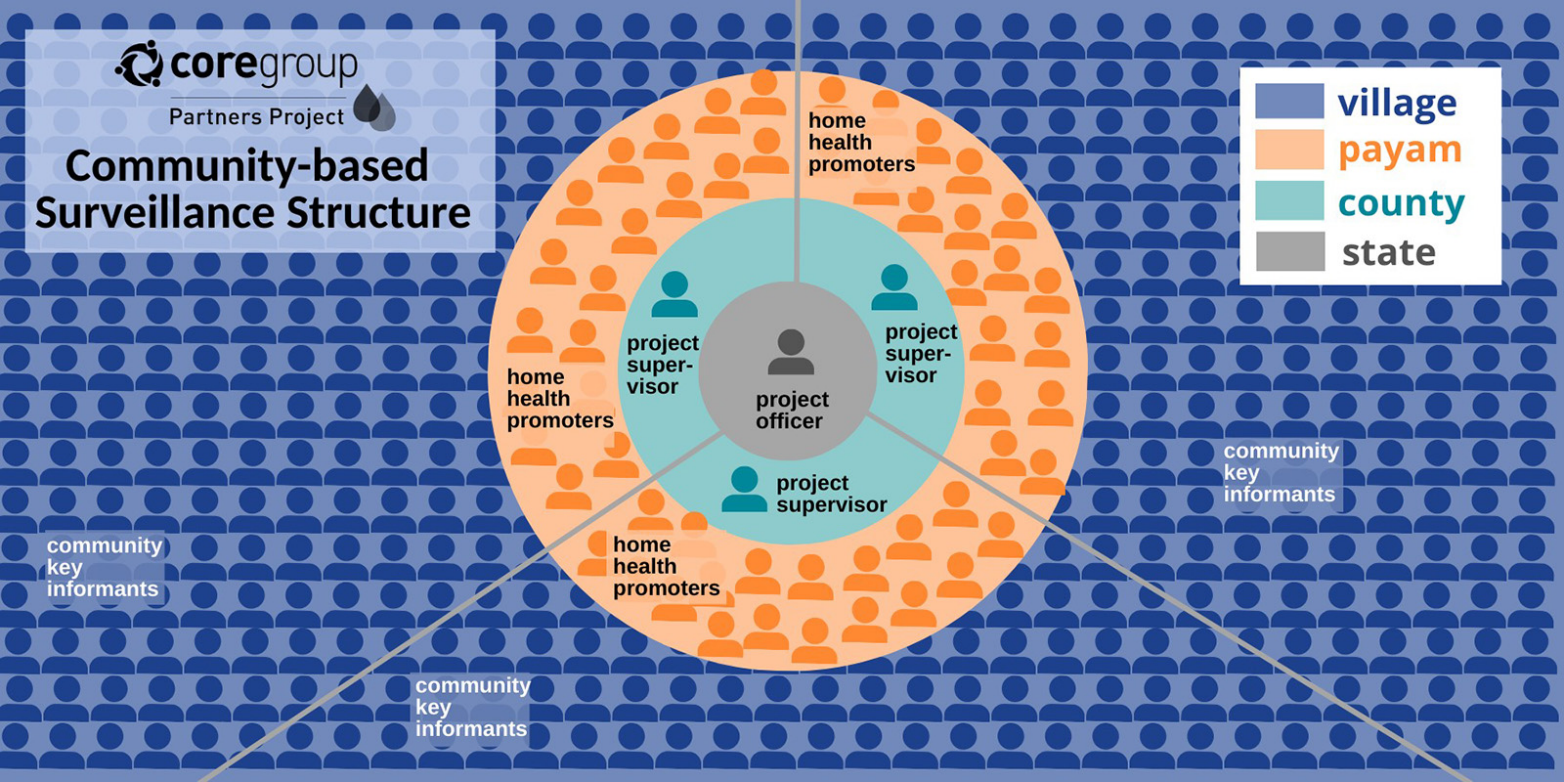
Community Structure of CORE Group Partner’s Project in South Sudan

CGPP operates through an extensive tiered community structure of supervised health workers and volunteers that provides a platform for community-based activities, including demand generation and mobilization of the community members for outreach immunization services for both RI and COVID-19 (Figure 2). In 2022, CGPP South Sudan had a total of 6 (1 female, 5 male) project officers, 29 (1 female, 28 male) project supervisors, 540 (122 female, 418 male) home health promoters (HHPs), and 5,094 (2,435 female, 2,659 male) community key informants. HHPs engage the community and provide RCCE through visits to homes and social places and mother-to-mother meetings. These activities are critical to immunization demand creation and social mobilization for outreach vaccination. HHPs are also integral to the CBS system, identifying and reporting suspected cases of priority diseases. Currently, the HHPs are not administratively linked to the government, though they are linked to the health facilities to facilitate the referral of cases. HHP selection is through their community leadership, chiefs, and council elders, who recommend individuals to serve. Community key informants are highly influential members of the community, such as teachers, religious leaders, and traditional healers, among others, who volunteer and are trained on community case definitions for priority diseases. They support timely detection of suspected disease cases and report these to HHPs. This network of health workers and volunteers has received training from CGPP and has a keen understanding of the working relationships between the communities and the health facilities. HHPs receive 3 days of training on basic integrated CBS, community case definitions for priority diseases, RCCE, social mobilization, and reporting, as well as on-the-job training through CGPP’s supportive supervision mechanism. The training and capacity-building aims to help HHPs continue to support their community and promote the government’s Boma Health Initiative. Community key informants receive regular mentorship through HHPs on community case definitions of priority diseases to promote early detection and referral. These individuals were also trained as social mobilizers to increase awareness of the COVID-19 vaccine during the COVID-19 vaccination campaigns. These established relationships with the community and the 2-way feedback mechanism are crucial for integration efforts.

When the first cases of COVID-19 were identified in South Sudan in March 2020, CGPP began providing its focal communities with clear, actionable information to reduce spread. To respond to the COVID-19 pandemic, CGPP received funding to integrate RCCE and CBS for COVID-19 prevention into messaging and activities for polio and other priority health conditions. After the introduction of the COVID-19 vaccine in South Sudan, CGPP received funding in April 2021 to improve COVID-19 vaccine uptake in project areas through social mobilization, additional RCCE, and outreach and mobile vaccination sessions. At the time of data collection for this article, only adults older than 18 years were eligible for the COVID-19 vaccine. With the urging of donors and the success of integrated RCCE and CBS, CGPP launched efforts to integrate COVID-19 vaccination and RI service delivery in April 2022. In addition to providing appropriate vaccination services for children and adults, these integrated outreach service delivery points offered other key primary health care services through partners. Other implementers of integrated RI and COVID-19 programs have found improved vaccination coverage as a result of integration.^21^ This article provides a blueprint for these integration approaches, laying out the steps CGPP followed, key considerations, results, and challenges of the integration. Coordination, training, outreach immunization services, CBS, and data management were all prioritized during the integration. This article also details the impact of the CGPP’s service delivery integration on vaccination coverage for both RI and COVID-19 vaccinations.

In April 2022, CGPP launched efforts to integrate COVID-19 vaccination and RI, as well as other key primary health care services.

## IMPLEMENTATION OF COVID-19 VACCINATION INTEGRATION

CGPP’s integration of COVID-19 vaccination for adults with RI for children was driven by the need to generate demand and improve uptake for both the COVID-19 vaccine and OPV, particularly in hard-to-reach communities. Integration provided an opportunity to use the infrastructure and human resources established for polio eradication to increase availability of information and make service delivery more accessible, cost effective, and efficient. One of the greatest challenges of service delivery before COVID-19 and within the COVID-19 response activities was the fragmentation of efforts by partners. In many cases, partners operated in silos, planning and delivering services without collaborating with others.

CGPP had experience with integrated service delivery within its previous efforts to implement interventions related to polio and VPDs alongside those for Ebola virus disease. CGPP leveraged the success of these program-specific integrated efforts to advocate for broader integration of service delivery by capitalizing on the programming and plans of partners working in other spheres like nutrition and reproductive health. For COVID-19, CGPP’s initial activities included integrated interventions, such as RCCE for multiple health topics and CBS. The project then added integrated service delivery through fixed vaccination sites, mobile vaccination sites, and mass vaccination campaigns, as explained in this article. When possible, these integrated service delivery sessions offered additional health and nutrition services, including nutrition education and food baskets, health screenings, antenatal care, and treatment of common diseases. These services were provided through county health departments by coordinated efforts among partner agencies and other nongovernmental organizations, including CGPP. Partners moved together in convoys to reach underserved populations, which proved to be the most effective strategy in high-security risk areas. Each provided services specific to their area of expertise. These integrated “one-stop shops” for vaccination saved time and money and increased access for community members, particularly women, who were under-vaccinated in the early days of the vaccine roll-out. In all 24 of CGPP’s focal counties, services were provided in one-stop shops for 7-day periods each month from April to September 2022 in hard-to-reach parts of CGPP’s implementation areas. The remaining 21 days each month were used for reporting and planning the next session. Planning was conducted based on data and other information from the implementation and special considerations for the ever-changing insecurity and weather/terrain context of the focal counties in South Sudan.

### Integration Phases

**FIGURE 3:**
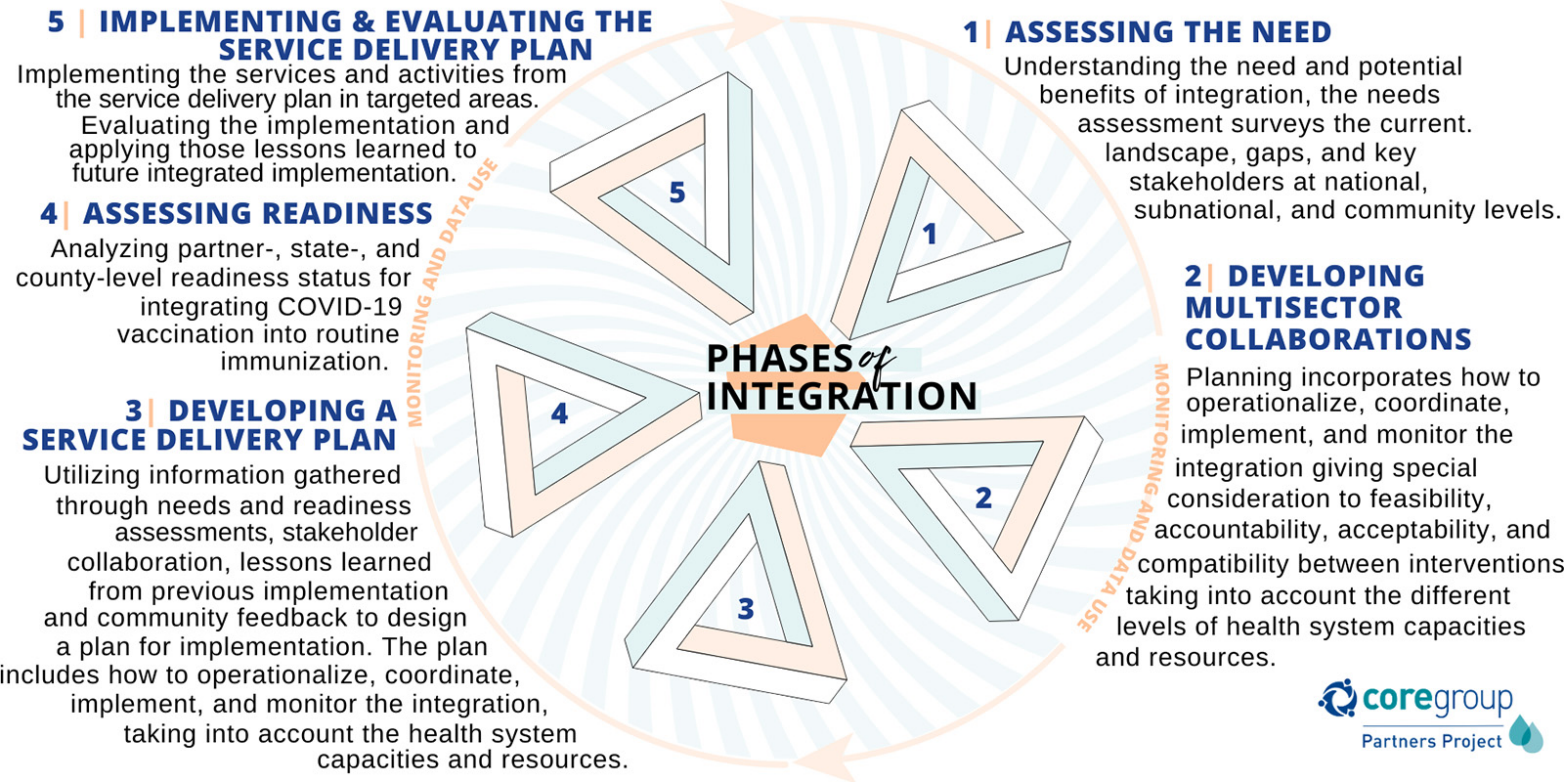
CORE Group Partner’s Project COVID-19 Vaccination and Routine Immunization Integration Phases in South Sudan

We summarize the phases used by CGPP and partners to deliver integrated health services (Figure 3). Although the phases outlined were followed sequentially for each specific example of integrated services delivery, it is critical to understand that some elements were also implemented in parallel. For example, as service delivery plans were developed, they were often changed and adapted as different needs arose based on additional multisectoral collaborations or community feedback. The process was constantly evolving. With each integrated service delivery session or each one-stop shop, new information was learned that changed the way the process worked for the next iteration.

#### Phase 1: Assessing the Need

The first step of planning integration was understanding the need and potential benefits of integration, the current landscape and gaps, and the stakeholders to engage. A needs assessment was conducted at different levels of the health and services delivery system. Additionally, key stakeholders and partners were identified at the national, subnational, and community levels.

At the national and subnational levels, needs assessments were conducted through consultative meetings during health coordination meetings with various partners, including the MOH, partner nongovernmental organizations, and international partners like WHO and UNICEF. These meetings included discussion and feedback on reporting tools and guidelines, vaccine availability, supply chain management, and information, education, and communication materials available for social mobilization. The needs assessment feedback was provided to stakeholders at all levels through health coordination meetings at county health departments, state MOHs, and the national COVID-19 task force.

At the state and county levels, the needs assessment focused on understanding the availability of vaccines, cold chain functionality and management, data management and reporting, health facility microplanning and mapping of communities, human resource availability, training needs on immunization in practice, and social mobilization. Findings from the needs assessments, including the capacities and resources at different levels of the health system, were vital to establishing a plan in phase 4.

At the community level, the needs assessment focused on understanding community needs and barriers, identifying priority communities, and exploring different methods to address barriers to immunization (e.g., physical barriers like insecurity, flooding, and distance; informational barriers including myths and misconceptions; and individual barriers like perceived efficacy and risk). This information was collected from communities through community dialogues, informal conversations, and volunteer observations. These assessments were critical to understanding why communities were not accessing services and identifying bottlenecks before planning.

#### Phase 2: Developing Multisector Collaborations

When integration efforts began, South Sudan’s national RI program and COVID-19 response program were independently functioning entities with little overlap. Each had its own technical working group, management and data systems, leadership and staff, and government mandates, creating challenges and opportunities. Partnership and collaboration were necessary for integrated service delivery to be successful. CGPP South Sudan was able to leverage collaboration and coordination mechanisms established through polio programming, including technical working groups (Expanded Program on Immunization technical working group, supplementary immunization activities subgroup, public health national steering committee, emergency preparedness and response technical working group, state MOH cluster meetings, and county cluster meetings), which brought together key players from different sectors. Multisectoral consultations between government entities, MOH immunization program leadership, county health departments, state MOHs, immunization and COVID-19 technical working groups, donors, and project implementing partners yielded plans and solutions that considered different viewpoints, used partner strengths, relied on different data points, and could better address the broader needs of communities.

#### Phase 3: Developing a Service Delivery Plan

Service delivery plans were working documents that were updated and refined. These plans had information on how to operationalize, coordinate, implement, and monitor the integration of COVID-19 vaccination and RI and emphasized the need and pathway to repurpose the existing polio workforce. Each program or partner developed the service delivery plans and presented them during weekly COVID-19 technical working group coordination meetings, where various partners, including WHO and UNICEF, and government entities, such as MOH officials, gave their feedback. During these meetings, CGPP advocated for the integration of partner service delivery. For example, if a partner planned to hold a nutrition screening, CGPP sent vaccinators and mobilizers to the screening and encouraged other partners to provide additional health services. CGPP contributed vaccinators, social mobilization, RCCE, and other technical and logistical support to the integrated efforts.

The development of a service delivery plan focused on individual and population-centered provision of packaged essential health services (assuming they were available) and assuring community participation and engagement. Special consideration was given to feasibility, accountability, compatibility between interventions, and acceptability to individuals, caregivers, health workers, and communities. The mandate was to determine how to reach the most marginalized, isolated, and unreached communities in rural and urban areas by combining COVID-19 vaccines with other essential health interventions through a trained, existing polio workforce of community health workers and volunteers.

Service delivery plans were established at the county level through collaboration, feedback, and input from a variety of stakeholders. Establishing plans at the county level allowed for flexibility and enabled each county to tailor the plan to the unique contextual factors, including their RI and COVID-19 vaccine coverage levels, health systems capacity, and overall progress toward integration. To develop the country service delivery plans, the following real-time data and information were used.

Integrated service delivery plans were established at the county level, allowing plans to be tailored to unique contextual factors, including vaccine coverage, health systems capacity, and overall progress toward integration.

- The proportion of COVID-19 high-risk populations (e.g., elderly, health care workers, and those with comorbid conditions)
- State and county COVID-19 vaccination performance
- Lessons learned from regional RI, COVID-19 vaccination, and previous efforts to integrate health services
- Health workforce competencies (including the prioritization of skilled vaccinators for the roll-out)
- Acceptability and perceptions of health workers and communities on integration
- Collaboration with other programs to link eligible individuals to other needed services (including consultation clinic/outreach, antenatal care, and food distribution)

Special consideration was given to service-user experience, demands on workforce, and workforce infection prevention and control training needs to successfully achieve linkages. Learnings and data from an integrated service delivery event were used to inform the implementation of the next service delivery event. For example, the proportion of the high-risk population was always changing and constantly updated due to the highly mobile nature of the population. Microplans were changed to account for new locations of the outreach sessions and the quantity of vaccines needed. Additional on-the-job training was provided when gaps were found, and messaging was changed based on the experiences of community participants.

#### Phase 4: Assessing Implementation Readiness

In all 24 implementation counties, CGPP conducted readiness assessments to analyze whether minimum standards of readiness for integration of COVID-19 vaccination with RI and implementation of service delivery plans were met at partner, state, and county levels. This analysis included conversations and collaborations with implementers, partners, and other key stakeholders at each health system level. The following 5 readiness indicators were tracked to determine the readiness.

1. Completion of microplanning alongside WHO, UNICEF, John Snow, Inc., and the national and subnational MOHs, including budgeting, monitoring and supervision plan, social mobilization plan, accessibility mapping plan, and vaccine management and monitoring plan
2. Validation of plans by the Expanded Program on Immunization/COVID-19 technical working groups and health coordination committees at the national, state, and county levels
3. Approval of plans and budgets by the state MOH and county health authorities
4. Completion of recruitment and training of vaccinators, social mobilizers, and supervisors to report adverse events following immunization
5. Completion of vaccine supply chain and cold chain plans from the state to the county and health facility level and a functional cold chain with working equipment at each level of the system
6. Adequate service delivery plan in place that details partner engagement, services provided, and timing

A main challenge was the development of microplans, which relied on key community leaders to provide data on the population and households within their village. Due to security challenges, many of the leaders stayed within safety zones, not in the communities they were from, making it difficult to estimate the population available, quantity of vaccines to be provided, and number of teams to be deployed.

#### Phase 5: Implementing and Evaluating Service Delivery Plan

Globally, the COVID-19 vaccine response was able to build upon the mechanisms and approaches developed for polio programming by using new tools, including real-time digital health monitoring systems, social listening mechanisms, short message service (SMS) reminders, and dashboards for visualization.^22^ These new tools were used to collect and monitor data and ensure that real-time data were used to improve subsequent implementation. CGPP devised a hybrid approach to vaccination service delivery using the 2 methods typically used to reach children with immunization—routine methods and mass campaigns—to allow for more opportunities for both children and adults to receive vaccinations. Both types of service delivery were accompanied by integrated RCCE messaging and social mobilization to ensure that communities had clear, actionable information about the benefits of vaccination. Throughout the integration phases, the provision of services and the experience of community participants were evaluated and used to inform the project and adjust strategies for more effective outcomes. For example, when parents raised concerns that children who were not eligible for the COVID-19 vaccine would be mistakenly vaccinated during integrated sessions, adjustments were made to the mobile service delivery sites to ensure the services for adults and children were separated.

Routine methods of delivery included the administration of both routine childhood immunizations and COVID-19 vaccines at health facilities, mobile or outreach sites, and through periodic intensification of RI approaches. Commonly in South Sudanese facilities, RI services for children and COVID-19 vaccinations for adults are delivered separately as part of designated primary care appointments. However, given the lack of accessible, well-stocked, and sufficiently staffed health centers, there is low utilization of primary health services, particularly among men and women of nonreproductive age who tend to have little engagement with preventive primary health care. Immunization (COVID-19 and routine childhood) and other health services are delivered by health facility staff to remote and hard-to-reach communities through single-day visits to a mobile/temporary outreach site, typically located 5–15 km from a fixed facility. Although these mobile efforts reach many hard-to-reach communities by making services more accessible, there are still communities that are unreached due to protracted violence, fighting, and geographical barriers (e.g., mountainous terrain with no access roads). CGPP offered immunization services concurrently with other community interventions, such as food distribution. Periodic intensification of RI includes country-specific packages of preventive services delivered through regular events, such as child health days. CGPP used these opportunities to provide COVID-19 vaccination for parents and other adults bringing children for vaccination.

Mass immunization campaigns are used to rapidly deliver vaccinations to large groups of people to increase immunization coverage as part of disease control, elimination, or eradication programs or in response to disease outbreaks. CGPP leveraged government-executed planned mass immunization campaigns for polio and measles and those planned for COVID-19 as opportunities to provide both RIs to children and COVID-19 vaccinations for adults.

### Points of Integration of COVID-19 Vaccination to RI

Integration efforts were focused on areas of need determined through collaborative planning and readiness assessments. The following specific components for integration implementation at the county and community levels included RCCE, CBS, training, last-mile delivery, service delivery, supportive supervision, and data collection and use.

- Developing clear, integrated RCCE methods and messaging that included key points on both polio/RI and COVID-19 vaccination and training existing polio project community health workers to deliver effectively to communities.
- Training health staff and vaccinators that included modules on polio/RI schedules and administration, COVID-19 administration, handling, standard operating procedures, and managing adverse effects following immunization.
- Using sample collection procedures, cold chains, and results provision mechanisms established for polio surveillance for rapid diagnostic and lab testing (COVID-19 tests were conducted at the health facility level, and the surveillance network conducted screening, sample collection, and transportation to the designated lab, especially from the point of entry at the borders).
- Establishing an integrated CBS system that could report suspected cases of polio and other VPDs, COVID-19, and adverse events after immunization.
- Integrating last-mile delivery of both COVID-19 and RI vaccines to the health facilities.
- Providing supportive supervision activities for COVID-19 and RI at integrated mobile service delivery points (supportive supervision was provided by CGPP, MOH, WHO, UNICEF, and John Snow, Inc.).
- Developing integrated monitoring tools, including reporting forms, supervision checklists, databases, and indicators for data collection and use.
- Integrating service delivery and providing both types of vaccination services at sites that previously only provided COVID-19 or RI.

Real-time monitoring was used throughout the process of integration to track progress of key indicators, allocate resources, and make implementation decisions. Monitoring data helped to identify areas where integration was making a key contribution and where interventions were not working as intended. Using real-time data allowed the project to make improvements and pivots as necessary. Real-time data were also integral to the allocation of human resources, vaccines, and supplies for vaccine sites and campaigns. These data were collected through MOH paper-based tools/registers and submitted electronically through ODK, which was then sent to the organizational network analysis aggregate server run by WHO. Trained recorders, payam supervisors, county monitoring and evaluation officers, and project supervisors collected and submitted these data.

## RESULTS: IMPACTS OF INTEGRATION

**FIGURE 4:**
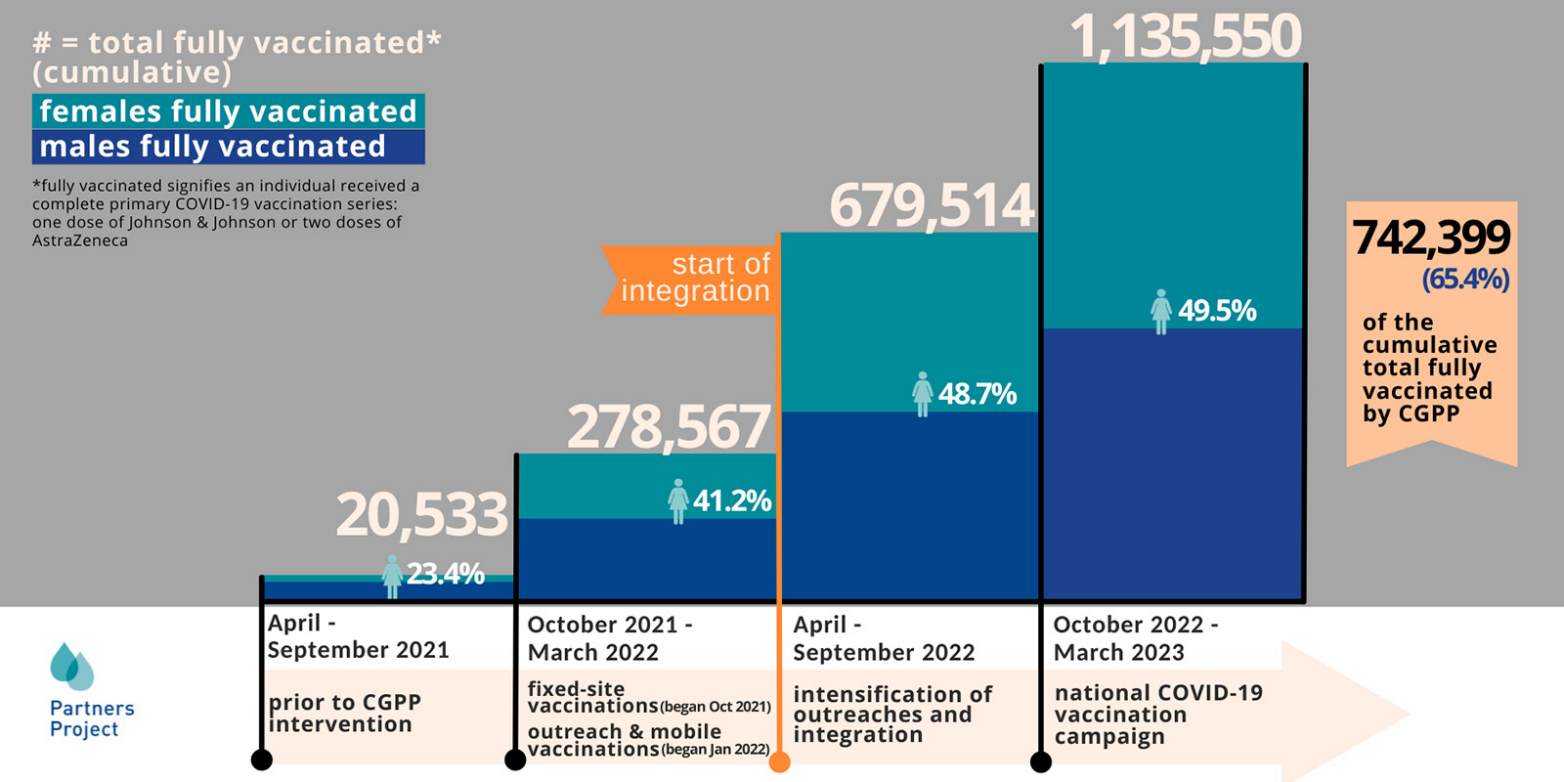
Cumulative Number of People Fully Vaccinated With COVID-19 in South Sudan, April 2021–March 2023 Abbreviation: CGPP, Core Group Partners Project.

**FIGURE 5:**
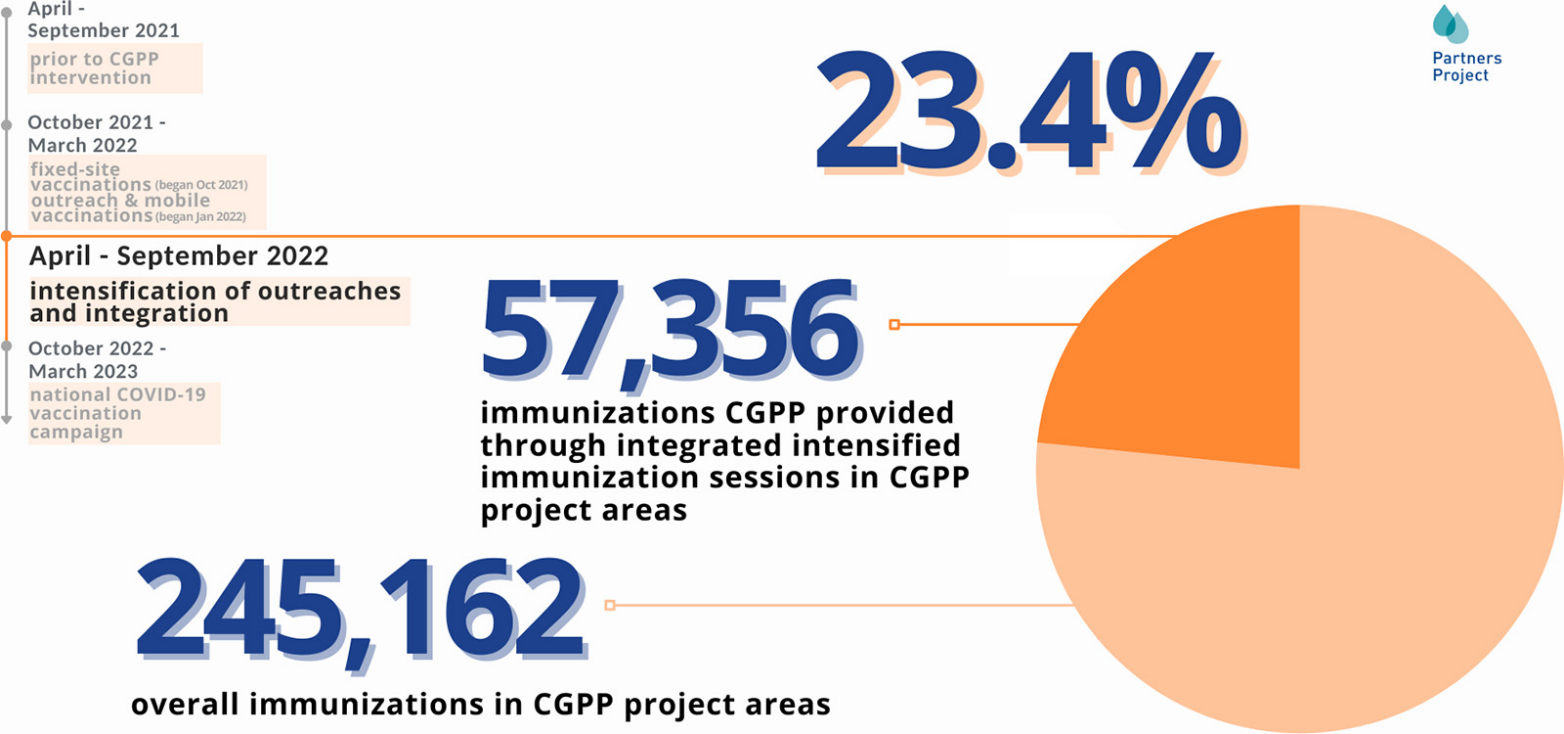
Total Routine Immunization Doses Given to Children Aged Younger Than 1 Year in CGPP Implementation Areas in South Sudan Abbreviation: CGPP, CORE Group Partners Project.

Integrated outreach and service delivery resulted in increased access to RI, COVID-19 vaccination, and other health services for children and adults. CGPP partnered with other agencies coordinating with county governments to implement one-stop shops—where both children and adults could access care—that increased the number of access points and made them easier to access by tailoring to the needs of the community. Mobile or fixed integrated vaccine options saved families time, money, and effort. Instead of visiting several health clinics or immunization locations, all family members could receive needed treatments at 1 access point. Integrating immunization reduced missed opportunities and allowed health professionals to provide more screenings, immunization defaulter tracing, referrals, and more nutrition services. Notably, RI coverage and COVID-19 vaccination coverage both improved after CGPP involvement and integrated outreach and campaign activities (Figure 4 and Figure 5).

Integrated outreach and service delivery resulted in increased access to RI, COVID-19 vaccination, and other health services for children and adults.

### COVID-19 Vaccination Uptake

The impact of offering integrated vaccination sessions is illustrated through the steep rise in COVID-19 vaccinations provided in the period after integration versus before. Figure 4 illustrates the number of people vaccinated for COVID-19 in CGPP South Sudan’s implementation areas during designated months and stages of programming. In October 2021, CGPP began fixed-site vaccinations, and in January 2022, the project added outreach and mobile site vaccination. In April 2022, integrated COVID-19 and RI outreach sessions began, followed by a national COVID-19 vaccination campaign in December 2022. The cumulative number of fully vaccinated adults aged older than 18 years in CGPP implementation areas rose from 278,567 before integration to 1,135,550 after integration. Of those vaccinated in these areas, 65.4% (742,399 people) were vaccinated by CGPP vaccinators. The rest were vaccinated by other partners and MOH vaccination efforts. Also notable is the improvement in uptake among females. Before CGPP’s intervention, less than one-quarter of vaccines were administered to females. After initial fixed and mobile site vaccinations were coupled with intense RCCE, female vaccine uptake rose to 41.2%. After integrated outreach and national integrated vaccination campaigns, uptake of vaccines was equal among males and females (49.5%). These significant improvements among females are likely the result of both intensified RCCE, which addressed myths and misconceptions that impacted female vaccination, particularly those related to fertility, and the increased accessibility of COVID-19 vaccines alongside RI, as women are usually the caregivers who bring children for RI.

### RI Uptake

CGPP’s integrated outreach efforts coincided with the closure of several health facilities in implementation areas during 2022, leaving many communities with even more limited access to RI at health facilities. This made the integrated one-stop shops even more important. For children aged younger than 1 year, CGPP provided RI, including OPV, measles, and Penta 3. The project also referred women of childbearing age for tetanus and diphtheria vaccine. Integrated outreach vaccination sessions (in convenient spaces including markets, farmlands, churches, and at social gatherings) were responsible for administering one-third of all RIs for children aged younger than 1 year in project implementation areas in fiscal year 2022 (October 1, 2021 through September 30, 2022), a notable achievement given the size, scope, and funding of the project (Figure 5).

Integrated intensified immunization sessions (one-stop shops) began in April 2022, adding adult COVID-19 vaccination and other services to RI offerings. CGPP provided a total of 57,356 RI doses to children aged younger than 1 year through these integrated intensified immunization sessions, nearly one-quarter of all RIs administered in CGPP implementation areas from April to September 2022 (Figure 5). This included 9,418 doses of OPV0, 15,160 doses of OPV3, 15,754 doses of Penta 3, and 17,024 doses of the measles vaccine. This is a notable contribution given that many other immunization sessions were being held during this period at health centers, MOH-sponsored clinics, and by other projects.

Applying the lessons learned through the previous integration activities, CGPP supported the March/April 2023 national measles follow-up campaign in South Sudan, facilitated the integration of other RIs during the campaign, and recruited 5,459 vaccinators. In addition to the measles vaccine, 154,742 doses of RIs (bacillus Calmette-Guerin, inactivated polio vaccine, OPV, and Penta 3) were administered to children and 36,413 doses of tetanus-diphtheria vaccine to pregnant women and those of childbearing age.

### Cost Savings: COVID-19 Vaccination Cost Through Integrated Vaccine Provision

The benefits of integration included the use of the same workforces, physical resources, supplies, and learnings, which led to increased efficiency and reduced costs. Integration diminished the need to have parallel systems and duplicative human and physical resources. For example, instead of staffing 2 outreach vaccination sites or needing 2 vehicles to transport vaccines to 2 outreach sites, the same staff and resources were leveraged for a single delivery site. Minimal training and costs were needed as a result of using already trained and established staff, health workers, and volunteers to add COVID-19 programming, as opposed to using a new untrained workforce, which would be costly and time consuming. The cost of supportive supervision, a mechanism where health workers and volunteers are supported through visits and on-the-job training, was cut in half, as all activities could be supervised at once.

As a result of integrating and using existing community-based networks for service delivery, the total cost per COVID-19 vaccine administered through CGPP’s integrated vaccine provision was US$4.70, which was calculated from the total cost incurred divided by the number of individuals vaccinated. This cost is specific to CGPP as it leveraged the existing polio structure and included human resources deployed, last-mile delivery, demand generation activities, and supportive supervision. The cost is significantly lower than the estimated total costs by other COVID-19 partners in South Sudan to administer doses, ranging from US$10 to more than US$22.^23^

## DISCUSSION

The COVID-19 vaccination response provided an opportunity to leverage vaccination investments and innovations made for polio and RI. The resources allocated to polio eradication have helped to strengthen struggling health systems, bolster cold chains and lab capabilities, train skilled volunteers and community health workers to deliver RCCE, develop new protocols and strategies for mass vaccination campaigns, and strengthen disease surveillance and outbreak response.^24^^–^^26^ The COVID-19 vaccine response was able to build upon this framework by using new tools, including real-time digital health monitoring systems, social listening mechanisms, SMS reminders, and dashboards for visualization.^22^ In CGPP focal counties, these improved infrastructures stand ready to tackle the next disease outbreak or pandemic.

The COVID-19 pandemic impacted the performance of immunization and other essential services in 2020 and 2021. Although there is evidence of recovery in many settings, others remain at a deficit.^27^ For South Sudan, the pandemic compounded challenges with RI delivery and the health system. Traditionally, immunization programs vaccinate children, adolescents, and women of reproductive age. With the introduction of the COVID-19 vaccine, countries like South Sudan were presented with the challenge of quickly vaccinating adult populations that don’t typically receive regular vaccinations.

Uptake was slow in the beginning, as myths and misconceptions plagued vaccine roll-out and caused vaccine hesitancy and fear. These were particularly hindering to women who feared the vaccine would impact current pregnancies and future fertility. For CGPP, harnessing the trusted relationships built over more than a decade of polio programming was integral to improving both COVID-19 vaccine and RI uptake in project areas. With clear linkages and understanding of community feedback, CGPP was quickly able to integrate RCCE messages for COVID-19 prevention and vaccination into already established channels of information transfer. Using trusted and trained community health workers allayed fears and addressed misconceptions.

The CGPP South Sudan experience provides support for the ability of polio-trained community health workers and volunteers to learn and apply new skills and information quickly. As countries look to polio eradication, the workforce and human resources stand ready to take on new challenges and should be harnessed to do so. At the service delivery level, integration requires greater capacity and responsibility from health care providers and community health workers.^28^ The potential burden on health workers who are often already overused, underpaid, and working under the constraints of conflict should also be considered. Future efforts should account for the human resources and capacity needs to alleviate any burnout potential. CGPP listened to volunteers, health workers, and vaccinators to try to address these concerns during service delivery and other programming. Additionally, the security situation in South Sudan presented a large challenge. CGPP constantly monitored the areas of implementation with security and logistics staff from partners and used informal channels to gather information from key informants and community gatekeepers to ensure that services were delivered safely.

Integration of service delivery provided another opportunity to use the infrastructure and systems created to deliver polio immunization through RI and mass vaccination campaigns. By blending these services and creating one-stop shops and outreach immunization sessions where parents and children could access services together, vaccine uptake improved. This outcome was in line with other studies, suggesting that immunization in South Sudan may be considerably enhanced by a combination of effective health education, easy access to health facilities, and utilization.^29^ Child health campaigns and outreach sessions had previously missed opportunities to provide service to adults. In this new model, families could reach key health services without multiple trips to different locations, cutting down on the time and expense needed to seek care. The results of CGPP’s integration activities supported another study that found that integrating immunization service delivery with other health services, such as ambulatory care for children and nutritional services, increased vaccination coverage, and reduced rates of children immunization schedule dropout in South Sudan.^30^ Additional benefits of health service integration and the use of shared resources included cost of health service delivery in a variety of settings, improved efficacy, and shorter time to initiation of services and treatment.^22^^,^^27^^,^^31^^–^^34^

Health program integration is not without challenges. When not carefully managed, integration can overshadow or divert resources from 1 key project area to another. CGPP experienced this at the beginning of the COVID-19 pandemic and during the initial stages of integration. County resources, including human resources initially earmarked for polio eradication and RI, were diverted to COVID-19 programming, contributing to declining RI. Program integration requires significant buy-in, collaboration, strong logistics, and coordinated planning from government workers, implementers, practitioners, volunteers, and communities. Without strong policy and commitment at each level of the health system, integration has the potential to strain already fragile health systems.^35^ To combat this, CGPP continued strong advocacy at the national and subnational levels to ensure that the prevention of polio and other VPDs stayed at the forefront of the COVID-19 response. CGPP also strengthened its supportive supervision to ensure that volunteers and health workers remained engaged and did not lose focus.

The lack of coordination and the separation of the polio eradication program and COVID-19 response at both the national and subnational levels makes coordination and collaboration difficult, particularly in South Sudan, where political will and policies have lagged behind. Advocacy for policies that support the use of integrated health delivery approaches is critical and should be a focus in international health communities and among donors. Without this framework, it will be difficult to bring stakeholders on board and get their buy-in.

Advocacy is necessary not only at the national and subnational levels but also at the community level. When rolling out integrated outreach sessions to deliver COVID-19 and RI simultaneously, CGPP received feedback that parents were not attending sessions because they feared that children would receive COVID-19 vaccines that had not yet been approved for children in South Sudan. Strong engagement with the community and constant rumor and myth monitoring through community health workers allowed CGPP to quickly address these barriers at vaccination sites. Separate tables for registration and vaccination were set up for children and adults to ease fears and dispel misconceptions. This example serves as a reminder for future efforts that communities should participate in the codesign of integrated immunization and health interventions that improve access and alleviate barriers to service uptake.

The investments made through COVID-19 response and integration—especially those to improve human resource capacity, immunization supply chain and logistics, digital tools, surveillance, data capture, and communications—have built upon the legacy of polio eradication efforts. These advances could continue to be leveraged and built upon to improve primary health care systems and access to a variety of health services for the most vulnerable communities. When considering integrated service delivery, particularly the integration of routine immunization and COVID-19 vaccination, the following recommendations should be considered.

### Recommendations

1. Integrated service delivery at the community level should be based on understanding the community’s needs, access issues, service gaps and barriers, and other key components of service uptake.
2. Integrated immunization and health programming should be codesigned with communities, incorporating community feedback to adjust and improve approaches that are not working.
3. Integration should not be solely limited to codelivery at the service level but must also include health governance functions, such as planning, program design, budgeting, and joint coordination under a single MOH department.
4. Policy changes and advocacy at the national government level are needed to support integration and allow for adequate funding, policy, and regulatory guidance. Political action and support can provide a strong framework for integration at all levels of the health system.
5. MOHs and health systems need to be intentional about breaking down silos and incentivizing multisectoral, multidisciplinary collaboration, partnership, and integration.
6. The potential burdens of integration on the health systems must be addressed, particularly the stress on health workers. Implementers must look for ways to address burnout, capacity gaps, and continued strain on health care workers, community health workers, and volunteers.
7. There must be continued work on resource mobilization and supply chain adaptation to ensure necessary supplies and assets are in place to support integrated systems.
8. Implementation research is needed to understand the impact and utility of different integration approaches.
9. The collection of monitoring data is paramount to success and should be used in making decisions, assessing integration implementation, and making course corrections.

## CONCLUSION

Integration of COVID-19 vaccination into polio and RI programs is possible and can yield exceptional results. However, it requires clear policy guidelines, commitment, and involvement of stakeholders at all levels to increase awareness, respond to misconceptions, and provide assurance to communities.

## Supplementary Material

GHSP-23-00178-Kisanga-article-summary_French.pdf

GHSP-23-00178-Kisanga-article-summary_English.pdf

GHSP-23-00178-Kisanga-article-summary_Portuguese.pdf

## Notes

**Peer Reviewed**

**First published online:** December 22, 2023.
