# Supplementary material for: Leveraging the CORE Group Partners Project Polio Infrastructure to Integrate COVID-19 Vaccination and Routine Immunization in South Sudan
Source: Glob Health Sci Pract. 2024 Feb 20;12(Suppl 1):e2300178. doi: 10.9745/GHSP-D-23-00178 (PMC10948123; doi:10.9745/GHSP-D-23-00178)
Supplement: GHSP-23-00178-Kisanga-article-summary_French.pdf [file GHSP-23-00178-Kisanga-article-summary_French.pdf]

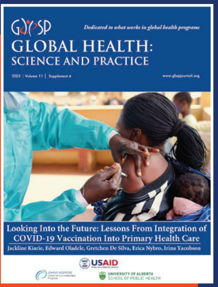

Tirer parti de l'infrastructure du Projet Polio des partenaires de la CORE Group pour intégrer la vaccination contre la COVID-19 et l'immunisation de routine au Soudan du Sud : Résumé de l'article

Anthony Kisanga, Kathy Vassos Stamidis, Samuel Rumbe, Doris Lamunu, Adil Ben, Gena Ruocco Thomas, Jean Berchmans

De quoi parle cet article ? Cet article décrit le processus du projet des partenaires de la CORE Group (CGPP) visant à intégrer les activités de vaccination contre la COVID-19 dans le cadre des efforts actuels d'éradication de la polio au Soudan du Sud et présente en détail les succès et les difficultés rencontrés, ainsi que l'impact sur la couverture des vaccinations de routine et des vaccinations contre la COVID-19.

Quels étaient les résultats ?
L'intégration des activités de vaccination contre la COVID-19 dans le processus d'éradication de la poliomyélite s'est concentrée sur la mise en œuvre au niveau des comtés et des communautés de plusieurs manières à savoir, la communication sur les risques et l'engagement communautaire, la formation du personnel de santé et des vaccinateurs, la surveillance communautaire, la livraison sur le dernier kilomètre, la prestation de services, la chaîne du froid et les tests de laboratoire, ainsi que la collecte et l'utilisation des données.

Ces efforts de mise en œuvre intégrés ont permis de réaliser des progrès ci-après :

- **Taux de vaccination contre la COVID-19 :** Le nombre d'adultes âgés de 18 ans et plus complètement vaccinés est passé d'environ 278 000 personnes en mars 2022 à plus de 1,1 million de personnes en mars 2023 après l'intégration. Le CGPP a administré 742 399 de ces vaccins dans le cadre de ses activités de sensibilisation.

Points de vue des auteurs

Le projet des partenaires du groupe CORE vise à renforcer les efforts des pays hôtes pour éradiquer la polio et d'autres maladies zoonotiques et évitables par la vaccination.

« Des plans de prestation de services intégrés ont été établis à l'échelle des comtés, ce qui permet d'adapter les plans aux facteurs contextuels uniques, y compris la couverture vaccinale, la capacité des systèmes de santé et les progrès globaux vers l'intégration. »

- Anthony Kisanga, directeur du secrétariat, CORE Group Partners Project, Soudan du Sud

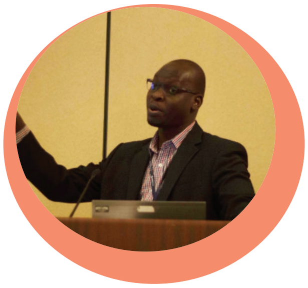

Lisez gratuitement l'article original publié dans Global Health Science and Practice à l'adresse suivante : https://doi.org/10.9745/GHSP-D-23-00178

Nombre de personnes entièrement vaccinées contre la COVID-19 au Soudan du Sud, avril 2021 -mars 2023

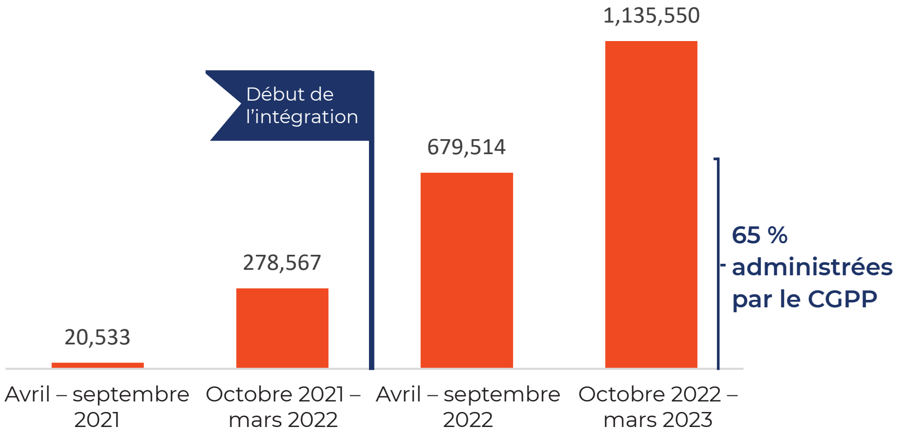

- **Couverture vaccinale de routine :** Entre avril et septembre 2022, 23 % des doses de vaccinations de routine administrées aux enfants de moins d'un an l'ont été dans le cadre d'activités intégrées du CGPP dans les zones de mise en œuvre du projet.

Doses totales de vaccinations de routine administrées aux enfants de moins d'un an dans les zones de mise en œuvre du CGPP

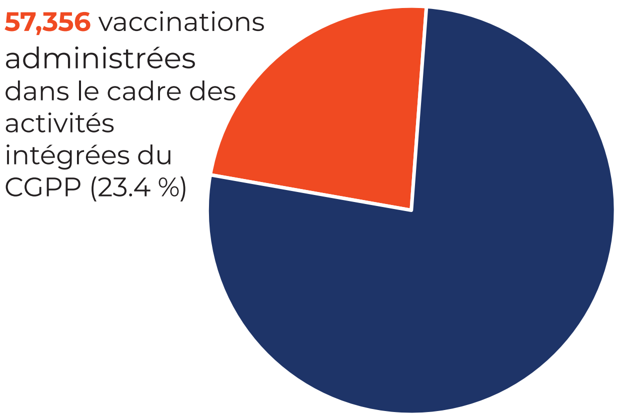

- **Réduction des coûts :** L'intégration des services a permis de réduire les coûts en partageant les effectifs, les ressources matérielles et les fournitures.

Le coût moyen par dose de vaccin contre la COVID-19 dans le cadre des activités intégrées était de 4,70 dollars, alors que les coûts pratiqués par d'autres partenaires au Soudan du Sud s'échelonnaient entre 10 et 22 dollars.

Le projet a connu plusieurs difficultés d'intégration, notamment en raison du détournement des ressources consacrées à l'éradication de la poliomyélite et à la vaccination de routine vers les efforts déployés dans le cadre du projet COVID-19. Ce problème a toutefois pu être surmonté grâce à la poursuite d'un plaidoyer vigoureux aux niveaux national et infranational pour que la poliomyélite et la vaccination systématique restent des priorités dans le cadre de la riposte à la COVID-19 et

pour garantir le soutien d'approches intégrées dans les politiques.

Des parents ne se présentaient pas aux séances de vaccination par crainte de voir leurs enfants recevoir des vaccins contre la COVID-19 non homologués pour les enfants. La solution a consisté à intensifier les efforts d'engagement communautaire et à lutter contre les rumeurs et les idées fausses.

Que signifient ces résultats ? L'intégration de la vaccination contre la COVID-19 à d'autres services de santé peut améliorer l'accès à la vaccination et réduire les coûts. Mais ce processus est difficile et nécessite des politiques claires, un engagement fort et la collaboration de nombreuses parties prenantes. L'utilisation de réseaux déjà fiables peut contribuer à lever les réticences à l'égard des vaccins et à limiter la duplication des efforts.

Pourquoi cette étude a-t-elle été réalisée ? La pandémie de la COVID-19 a perturbé les services de vaccination de routine au Soudan du Sud. Parallèlement, le pays a dû trouver de nouveaux moyens d'atteindre les adultes avec le vaccin contre la COVID-19 malgré les réticences et les obstacles logistiques.

Quand et où ces activités ont-elles été mises en œuvre ? Ces activités intégrées se sont déroulées au Soudan du Sud entre 2021 et 2023.

USAID FROM THE AMERICAN PEOPLE

Knowledge SUCCESS

Ce guide a été réalisé grâce au soutien du peuple américain par l'intermédiaire de l'Agence américaine pour le développement international dans le cadre du projet Knowledge SUCCESS (Strengthening Use, Capacity, Collaboration, Exchange, Synthesis, and Sharing, accord de coopération n° 7200AA19CA00001 avec Johns Hopkins University. Knowledge SUCCESS est soutenu par le Bureau de la santé mondiale de l'USAID, le Bureau de la population et de la santé reproductive, et dirigé par le John Hopkins Centre for Communications Programs (CCP) en partenariat avec Amref Health Africa, le Centre d'économie comportementale de Busara et « FHI 360 ». Les informations fournies dans cette ressource relèvent de la seule responsabilité de Knowledge SUCCESS et ne reflètent pas nécessairement les opinions de l'USAID, du gouvernement américain ou de Johns Hopkins University.
