# Supplementary material for: Leveraging the CORE Group Partners Project Polio Infrastructure to Integrate COVID-19 Vaccination and Routine Immunization in South Sudan
Source: Glob Health Sci Pract. 2024 Feb 20;12(Suppl 1):e2300178. doi: 10.9745/GHSP-D-23-00178 (PMC10948123; doi:10.9745/GHSP-D-23-00178)
Supplement: GHSP-23-00178-Kisanga-article-summary_English.pdf [file GHSP-23-00178-Kisanga-article-summary_English.pdf]

Lessons from Integration of COVID-19 Vaccination Into Primary Health Care | Article Summary Series

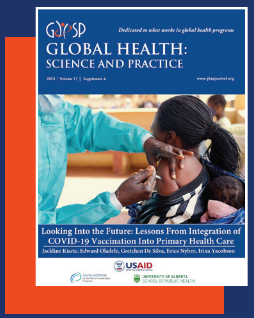

Leveraging the CORE Group Partners Project Polio Infrastructure to Integrate COVID-19 Vaccination and Routine Immunization in South Sudan: Article Summary

Anthony Kisanga, Kathy Vassos Stamidis, Samuel Rumbe, Doris Lamunu, Adil Ben, Gena Ruocco Thomas, Jean Berchmans

What is this article about? This article describes the process of the CORE Group Partners Project (CGPP) integrating COVID-19 vaccination activities with ongoing polio eradication efforts in South Sudan and details the successes and challenges, as well as its impact on the coverage of routine immunizations and COVID-19 vaccinations.

What were the results? Integration of COVID-19 vaccination with polio eradication efforts focused on integrating implementation at the county and community levels through: risk communication and community engagement, training health staff and vaccinators, community-based surveillance, last-mile delivery, service delivery, cold chain and lab testing, and data collection and use.

These integrated implementation efforts resulted in improved:

- **COVID-19 vaccination uptake**: The number of fully vaccinated adults aged 18 years and older increased from about 278,000 people in March 2022 to over 1.1 million by March 2023 after integration. CGPP administered 742,399 of these vaccinations through outreach efforts.

Number of People Fully Vaccinated with COVID-19 Vaccine in South Sudan, April 2021-March 2023

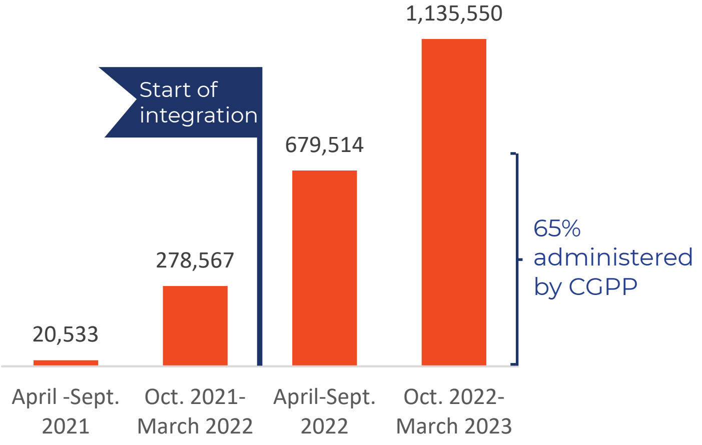

Insights from the Authors

The CORE Group Partners Project works to strengthen host country efforts to eradicate polio and other vaccine-preventable and zoonotic diseases.

"Integrated service delivery plans were established at the county level, allowing plans to be tailored to unique contextual factors, including vaccine coverage, health systems capacity, and overall progress toward integration."

- Anthony Kisanga, Secretariat Director, CORE Group Partners Project, South Sudan

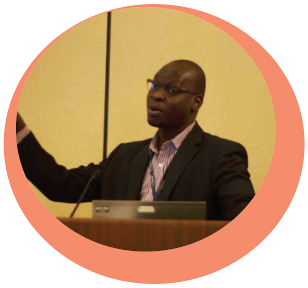

Read the original article published in Global Health Science and Practice for free at: https://doi.org/10.9745/GHSP-D-23-00178

- **Routine immunization coverage**: From April to September 2022, 23% of routine immunization doses given to children younger than one year were provided through integrated CGPP activities in project implementation areas.

Total Routine Immunization Doses Given to Children Under 1 Year in CGPP Implemenatation Areas

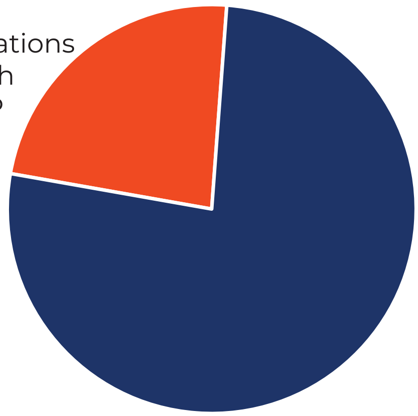

- **Cost savings:** Integrated service delivery reduced costs by sharing workforces, physical resources, and supplies.

The average cost per COVID-19 vaccine through the integrated activities was $4.70 compared to costs by other partners in South Sudan ranging from $10-$22.

Several challenges of integration that the project experienced included resources from polio eradication and routine immunization were diverted toward COVID-19 efforts. This was overcome by continuing strong advocacy at the national and subnational levels to ensure that polio and routine immunization remained priorities during the COVID-19 response and that policies support integrated approaches.

Some parents weren't attending vaccination sessions because of fears their children would receive COVID-19 vaccines that weren't approved for children. This was addressed by increasing community engagement efforts and combating rumors and misconceptions.

What do these results mean? Integration of COVID-19 vaccination and other health services can improve access and reduce costs. But this process is difficult and requires clear policies, strong commitment, and collaboration. from many stakeholders. Leveraging already trusted networks can help to address vaccine hesitancy, and limit duplication of efforts.

Why was this study done? The COVID-19 pandemic disrupted routine immunization services in South Sudan. At the same time, the country had to find new ways of reaching adults with the COVID-19 vaccine despite hesitancy and logistical barriers.

When and where were these activities implemented? These integrated activities took place in South Sudan between 2021 and 2023.

USAID FROM THE AMERICAN PEOPLE
Knowledge SUCCESS
This summary brief is made possible by the support of the American People through the U.S. Agency for International Development under the Knowledge SUCCESS (Strengthening Use, Capacity, Collaboration, Exchange, Synthesis, and Sharing) Project Cooperative Agreement No. 7200AA19CA00001 with the Johns Hopkins University.
Knowledge SUCCESS is supported by USAID's Bureau for Global Health, Office of Population and Reproductive Health and led by the Johns Hopkins Center for Communication Programs (CCP) in partnership with Amref Health Africa, The Busara Center for Behavioral Economics (Busara), and FHI 360. The information provided in this summary brief are the sole responsibility of Knowledge SUCCESS and does not necessarily reflect the views of USAID, the U.S. Government, or the Johns Hopkins University.
