# Supplementary material for: Leveraging the CORE Group Partners Project Polio Infrastructure to Integrate COVID-19 Vaccination and Routine Immunization in South Sudan
Source: Glob Health Sci Pract. 2024 Feb 20;12(Suppl 1):e2300178. doi: 10.9745/GHSP-D-23-00178 (PMC10948123; doi:10.9745/GHSP-D-23-00178)
Supplement: GHSP-23-00178-Kisanga-article-summary_Portuguese.pdf [file GHSP-23-00178-Kisanga-article-summary_Portuguese.pdf]

Lições da integração da vacinação contra a COVID-19 nos cuidados de saúde primários | Série de resumos de artigos

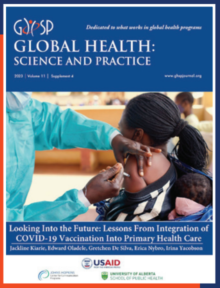

Alavancar a infraestrutura da poliomielite do Projeto de Parceiros do CORE Group para integrar a vacinação contra a COVID-19 e a imunização de rotina no Sudão do Sul: resumo do artigo

Anthony Kisanga, Kathy Vassos Stamidis, Samuel Rumbe, Doris Lamunu, Adil Ben, Gena Ruocco Thomas, Jean Berchmans

De que trata este artigo? Este artigo descreve o processo do Projeto de Parceiros do CORE Group (CGPP) que integra as atividades de vacinação contra a COVID-19 com os esforços de erradicação da poliomielite em curso no Sudão do Sul, e descreve em pormenor os sucessos e desafios, bem como o seu impacto na cobertura das imunizações de rotina e das vacinações contra a COVID-19.

Quais foram os resultados? A integração das atividades de vacinação contra a COVID-19 com os esforços de erradicação da poliomielite centrou-se na integração da implementação ao nível do condado e das comunidades de várias formas: comunicação de riscos e envolvimento da comunidade, formação de profissionais de saúde e dos vacinadores, vigilância baseada na comunidade, entrega na reta final, prestação de serviços, cadeia de frio e testes laboratoriais, e recolha e utilização de dados.

Estes esforços integrados de implementação resultaram em melhorias:

- **Adesão à vacinação contra a COVID-19:** o número de adultos com 18 anos ou mais totalmente vacinados aumentou de cerca de 278.000 pessoas em março de 2022 para mais de 1,1 milhões de pessoas em março de 2023 após a integração. O CGPP administrou 742.399 destas vacinas através de ações de sensibilização.

Perspetivas dos autores

O Projeto de Parceiros do CORE Group visa reforçar os esforços dos países de acolhimento para erradicar a poliomielite e outras doenças zoonóticas e passíveis de prevenção por vacinação.

"Os planos de prestação de serviços integrados foram estabelecidos ao nível dos condados, permitindo que fossem adaptados a fatores contextuais únicos, incluindo a cobertura vacinal, a capacidade dos sistemas de saúde e o progresso geral no sentido da integração."

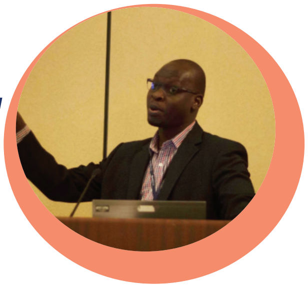

- Anthony Kisanga, Diretor do Secretariado, CORE Group Partners Project, Sudão do Sul

Leia o artigo original publicado na Global Health Science and Practice gratuitamente em:
https://doi.org/10.9745/GHSP-D-23-00178

Número de pessoas totalmente vacinadas com a vacina contra a COVID-19 no Sudão do Sul, abril de 2021 - março de 2023

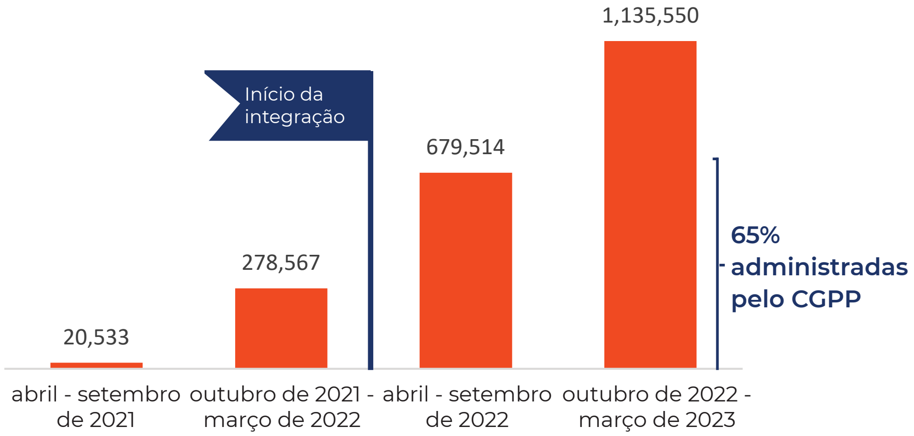

- **Cobertura da imunização de rotina:** de abril a setembro de 2022, 23% das doses de imunização de rotina administradas a crianças com menos de um ano foram fornecidas através de atividades integradas do CGPP nas áreas de implementação do projeto.

Total de doses de imunização de rotina administradas a crianças com menos de 1 ano nas áreas de implementação do CGPP

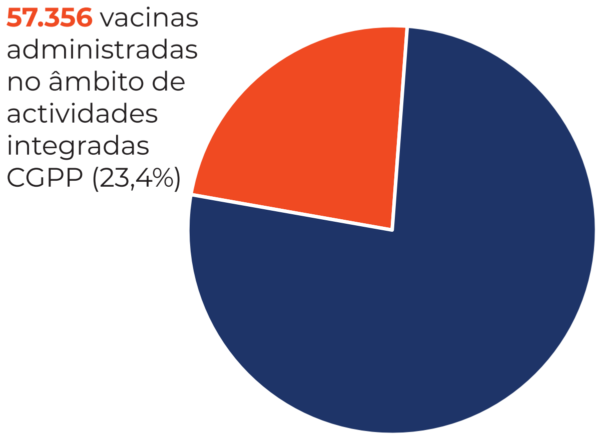

- **Redução de custos:** a prestação de serviços integrados reduziu os custos através da partilha de mão de obra, recursos físicos e fornecimentos.

O custo médio por vacina contra a COVID-19 através das atividades integradas foi de 4,70 dólares, em comparação com os custos de outros parceiros no Sudão do Sul, que variam entre 10 e 22 dólares.

Vários desafios de integração que o projeto enfrentou incluíram recursos da erradicação da poliomielite e da imunização de rotina que foram desviados para os esforços contra a COVID-19. Isto foi ultrapassado através da continuação de uma forte sensibilização aos níveis nacional e subnacional para garantir que a poliomielite e a imunização de rotina continuavam a ser prioridades durante a resposta à COVID-19, e que as políticas apoiavam abordagens integradas.

Alguns pais não compareciam às sessões de vacinação devido ao receio de que os seus filhos recebessem vacinas contra a COVID-19 que não tivessem sido aprovadas para crianças. Esta questão foi abordada através do aumento dos esforços de envolvimento da comunidade e do combate a rumores e ideias erradas.

O que significam estes resultados?
A integração da vacinação contra a COVID-19 e de outros serviços de saúde pode melhorar o acesso à vacinação e reduzir os custos. Mas este processo é difícil e exige políticas claras, um forte empenho e a colaboração de muitas partes interessadas. A utilização de redes de confiança já existentes pode ajudar a resolver a hesitação em vacinar e a limitar a duplicação de esforços.

Porque é que este estudo foi realizado?
A pandemia de COVID-19 perturbou os serviços de imunização de rotina no Sudão do Sul. Ao mesmo tempo, o país teve de encontrar novas formas de chegar aos adultos com a vacina contra a COVID-19, apesar da hesitação e das barreiras logísticas.

Quando e onde foram implementadas estas atividades? Estas atividades integradas tiveram lugar no Sudão do Sul entre 2021 e 2023.

USAID
FROM THE AMERICAN PEOPLE
Knowledge SUCCESS
Este guia resumido é possível graças ao apoio do povo americano através da U.S. Agency for International Development ao abrigo do Acordo de Cooperação do Projeto Knowledge SUCCESS (Strengthening Use, Capacity, Collaboration, Exchange, Synthesis, and Sharing) n.º 7200AA19CA00001 com a Universidade Johns Hopkins. O Knowledge SUCCESS é apoiado pelo Gabinete de Saúde Global, da População e Saúde Reprodutiva da USAID e dirigido pelo Johns Hopkins Center for Communication Programs (CCP) em parceria com a Amref Health Africa, The Busara Center for Behavioral Economics (Busara) e a FHI 360. As informações fornecidas neste guia resumido são da exclusiva responsabilidade da Knowledge SUCCESS e não refletem necessariamente as opiniões da USAID, do Governo dos EUA ou da Universidade Johns Hopkins.
